# Dual‐layer spectral CT for proton, helium, and carbon ion beam therapy planning of brain tumors

**DOI:** 10.1002/acm2.13465

**Published:** 2021-11-01

**Authors:** Friderike K. Longarino, Thomas Tessonnier, Stewart Mein, Semi B. Harrabi, Jürgen Debus, Wolfram Stiller, Andrea Mairani

**Affiliations:** ^1^ German Cancer Research Center (DKFZ) Clinical Cooperation Unit Radiation Oncology Heidelberg Germany; ^2^ Department of Radiation Oncology Heidelberg University Hospital Heidelberg Germany; ^3^ Department of Physics and Astronomy Heidelberg University Heidelberg Germany; ^4^ Heidelberg Ion Beam Therapy Center (HIT) Heidelberg Germany; ^5^ German Cancer Research Center (DKFZ) Translational Radiation Oncology Heidelberg Germany; ^6^ National Center for Radiation Research in Oncology (NCRO) Heidelberg Institute of Radiation Oncology (HIRO) Heidelberg Germany; ^7^ National Center for Tumor Diseases (NCT) Heidelberg Germany; ^8^ Partner Site Heidelberg German Cancer Consortium (DKTK) Heidelberg Germany; ^9^ Diagnostic and Interventional Radiology (DIR) Heidelberg University Hospital Heidelberg Germany; ^10^ Medical Physics National Centre of Oncological Hadrontherapy (CNAO) Pavia Italy

**Keywords:** brain tumors, dual‐layer spectral CT, ion beam therapy planning, range uncertainties, stopping power

## Abstract

Pretreatment computed tomography (CT) imaging is an essential component of the particle therapy treatment planning chain. Treatment planning and optimization with charged particles require accurate and precise estimations of ion beam range in tissues, characterized by the stopping power ratio (SPR). Reduction of range uncertainties arising from conventional CT‐number‐to‐SPR conversion based on single‐energy CT (SECT) imaging is of importance for improving clinical practice. Here, the application of a novel imaging and computational methodology using dual‐layer spectral CT (DLCT) was performed toward refining patient‐specific SPR estimates.

A workflow for DLCT‐based treatment planning was devised to evaluate SPR prediction for proton, helium, and carbon ion beam therapy planning in the brain. DLCT‐ and SECT‐based SPR predictions were compared in homogeneous and heterogeneous anatomical regions. This study included eight patients scanned for diagnostic purposes with a DLCT scanner. For each patient, four different treatment plans were created, simulating tumors in different parts of the brain.

For homogeneous anatomical regions, mean SPR differences of about 1% between the DLCT‐ and SECT‐based approaches were found. In plans of heterogeneous anatomies, relative (absolute) proton range shifts of 0.6% (0.4 mm) in the mean and up to 4.4% (2.1 mm) at the distal fall‐off were observed. In the investigated cohort, 12% of the evaluated organs‐at‐risk (OARs) presented differences in mean or maximum dose of more than 0.5 Gy (RBE) and up to 6.8 Gy (RBE) over the entire treatment. Range shifts and dose differences in OARs between DLCT and SECT in helium and carbon ion treatment plans were similar to protons.

In the majority of investigated cases (75th percentile), SECT‐ and DLCT‐based range estimations were within 0.6 mm. Nonetheless, the magnitude of patient‐specific range deviations between SECT and DLCT was clinically relevant in heterogeneous anatomical sites, suggesting further study in larger, more diverse cohorts. Results indicate that patients with brain tumors may benefit from DLCT‐based treatment planning.

## 1 INTRODUCTION

Radiotherapy using proton and light ion beams enables accurate and precise delivery of highly conformal dose distributions to the target volume while sparing normal tissues compared with conventional photon‐based radiotherapy.^1^, ^2^ To properly exploit these physical characteristics, clinical application imposes high accuracy requirements in treatment planning and delivery.^3^

Successful treatment planning and optimization require precise estimations of the energy deposited along the penetration path and the finite beam range of charged particles, characterized by the stopping power ratio relative to water (SPR), to model radiation transport and interactions within a patient.^3^ Pretreatment computed tomography (CT) imaging, providing anatomical and quantitative information for treatment planning, is an essential component of the radiotherapy treatment chain, and a topic of growing importance in ion beam therapy with relation to uncertainties in range prediction.^4^, ^5^ Compared with conventional photon‐based therapy, the conversion of CT numbers to the relevant physical quantities for dose calculation within a treatment planning system (TPS) (i.e., relative electron density (ED) or SPR) is more critical in particle therapy due to the high precision required to predict the Bragg peak position.^5^, ^6^ Today, standard clinical protocols involve image data acquisition with single‐energy CT (SECT) systems.^7^ However, clinical treatment planning with SECT‐based systems may be vulnerable to range prediction uncertainties due to generalized CT‐number‐to‐SPR conversion, lacking patient‐specificity,^6^, ^8^, ^9^ with uncertainties reaching up to 3.5% between planned and delivered beam range.^6^, ^9^, ^10^

Uncertainties in particle range prediction are considered via incorporation of safety margins during treatment planning, e.g., via the robust optimization concept. For clinical CT‐number‐to‐SPR calibration curves, also denoted Hounsfield look‐up tables (HLUTs), there is no one‐to‐one correlation (i.e., bijection) between CT numbers and SPRs.^8^ More specifically, two different materials with different compositions and physical properties (i.e., SPR) can exhibit identical CT numbers in SECT‐based images and vice versa.^9^ This nonbijectivity may be a source of systematic error between treatment planning and delivery^5^ leading to enlarged margins and compromising the advantages of particle therapy over photon radiotherapy.

To mitigate the highlighted uncertainties arising during particle therapy treatment planning, dual‐energy CT (DECT) systems, for acquisitions of two CT scans with different X‐ray spectra, are becoming increasingly available and potentially offer an improved SPR prediction in the clinic^7^, ^11^, ^12^, ^13^, ^14^ by making use of material‐specific and/or material density images.^7^, ^15^ Since the clinical introduction of a first‐generation dual‐source CT system for diagnostic imaging,^16^ a multitude of research studies identified various promising applications of DECT within the entire radiotherapy chain from tumor staging to delineation, tumor and normal tissue characterization, and dose calculations.^7^

Among the DECT acquisition methods available today, dual‐layer spectral CT (DLCT), an approach combining a single X‐ray source with a dual‐layer detector, has been recently introduced into clinical practice.^17^ Using two scintillator layers with different spectral sensitivities, DLCT enables simultaneous detection of two different energy levels for spectral imaging purposes, without the need to preselect specific CT protocols (e.g., different tube voltages).^18^ In turn, more comprehensive image data acquisition and quantification regarding material compositions in the human body is feasible compared with SECT‐based methods. Application of DLCT imaging and mathematical formalisms can yield direct patient‐specific determination of SPR maps, which, in turn, may lead to improved agreement between planned and delivered ion beam treatments as opposed to indirect SECT‐based SPR prediction. Moreover, unlike other published DECT‐based methods, SPR prediction using DLCT imaging enables projection‐based reconstruction^5^ and directly makes use of the physical quantities ED and effective atomic number (EAN) provided by the DLCT scanner as spectral output data (without any need for further calibrations or parametrizations) as input data for SPR prediction via the Bethe equation.

Substantial efforts have outlined and established treatment planning with other DECT acquisition methods^19^; however, to date, no study has presented investigations on the clinical feasibility of DLCT‐based treatment planning from the perspective of patient delivery. Notably, the impact and comparison of using DLCT for treatment planning with different ions, such as helium (^4^He) and carbon (^12^C) ions, in addition to protons (^1^H) have not yet been investigated in the literature. Prior to the clinical translation, an established clinical workflow and reliable benchmarks by means of quantitative DLCT imaging are needed for proper assessment. Previous works available in the literature present preclinical studies, illustrating the methodological development of SPR prediction with DLCT and experimental verification of the developed approach using tissue surrogates and anthropomorphic phantoms.^18^, ^20^, ^21^, ^22^, ^23^ The data suggest a mean DLCT‐based SPR prediction accuracy of 0.6% compared with measured SPR and 1 mm proton range prediction improvement in an anthropomorphic head phantom compared with SECT.^20^ Nevertheless, thorough investigations on how these improvements affect the dose distribution in patients, as well as identification of which patient subgroups would benefit the most from DLCT, have yet to be performed.

This study aims to investigate DLCT imaging for proton, helium, and carbon ion beam range prediction in brain tumors. A clinical workflow for DLCT‐based treatment planning is devised at the Heidelberg Ion Beam Therapy Center (HIT, Germany). Quantitative differences between SECT‐ and DLCT‐based SPR prediction (interpatient and intrapatient) are assessed in various clinical scenarios. Furthermore, SPR prediction performance is evaluated to identify clinical cases that benefit from DLCT‐based treatment planning in proton, helium, and carbon ion beam therapy.

## 2 METHODS

### 2.1 Patient cohort

The feasibility and accuracy of DLCT‐based particle therapy planning were investigated in a group of eight randomly selected diagnostic radiological patients (age, 28–85 years) by analyzing previously acquired (i.e., for diagnostic purposes) DLCT image data of the head. Head cases were chosen for the investigation for two reasons: (i) They contain both a variety of homogeneous and heterogeneous anatomical treatment sites important for testing different clinical conditions, and the majority of patients at the HIT facility are treated for brain cancers and head and neck cancers. (ii) Image data acquired with a CT image acquisition and reconstruction protocol similar to that used for ion beam therapy planning at HIT were available only for head cases. More specifically, all other data sets were acquired with either contrast agent or exposure modulation, which prevents their use in this treatment planning study.

DLCT is not yet implemented in the clinical routine for ion beam therapy treatment planning at our institution. Therefore, to explore the DLCT modality, image data for patients who have undergone diagnostic procedures using the DLCT scanner were analyzed. Subsequently, DLCT image data were retrospectively derived on the IQon Spectral CT IntelliSpace Portal workstation. All imaging with the DLCT scanner was performed for clinical indications; hence, no scan was conducted explicitly for the purpose of this study. Anonymized patient records were obtained with informed consent following the Declaration of Helsinki. Clearance from the ethical review committee was not required for the retrospective nature of the study.

### 2.2 Dual‐layer spectral CT imaging technique

The DLCT imaging technique (IQon Spectral CT, Philips Healthcare, Best, The Netherlands) is based on two detector layers with different spectral sensitivities that detect high‐ and low‐energy data simultaneously in time and space.^17^ Low‐energy photons from the X‐ray spectrum are selectively absorbed by the top layer yttrium‐based garnet scintillator, whereas high‐energy photons pass through the top layer and are absorbed by the bottom layer gadolinium oxysulfide scintillator.^17^ As a result, direct generation of quantitative spectral information (i.e., ED and EAN) is made possible on the full standard field‐of‐view of 500 mm for all performed scans, without the need of additional acquisitions or specific CT imaging protocols.^21^ Such methods using spectral data allow determining volumetric SPR maps that are patient‐specific and do not depend on generic CT‐number‐to‐SPR conversions.^20^

### 2.3 Image acquisition settings and reconstruction parameters

Image acquisitions were performed using the clinical protocol for adult head CT scans for diagnostics at our facility. The following acquisition settings were used: tube voltage of 120 kV_p_, tube current‐time product of 281 mAs (tube current modulation was deactivated), collimation of 64 × 0.625 mm, rotation time of 0.75 s, pitch of 0.39, CTDI_vol_ of 48.1 mGy, slice thickness of 2 mm, and slice spacing of 1.5 mm. The reconstruction filter UB, a spectral level, and the hybrid‐iterative reconstruction algorithm at iDose^4^ level (scale: 0–6) of 3 were used. For each patient scan, on the IQon Spectral CT scanner, both a SECT and DLCT data set were derived.

### 2.4 Methodology for performing DLCT data‐based SPR prediction and treatment planning

**FIGURE 1:**
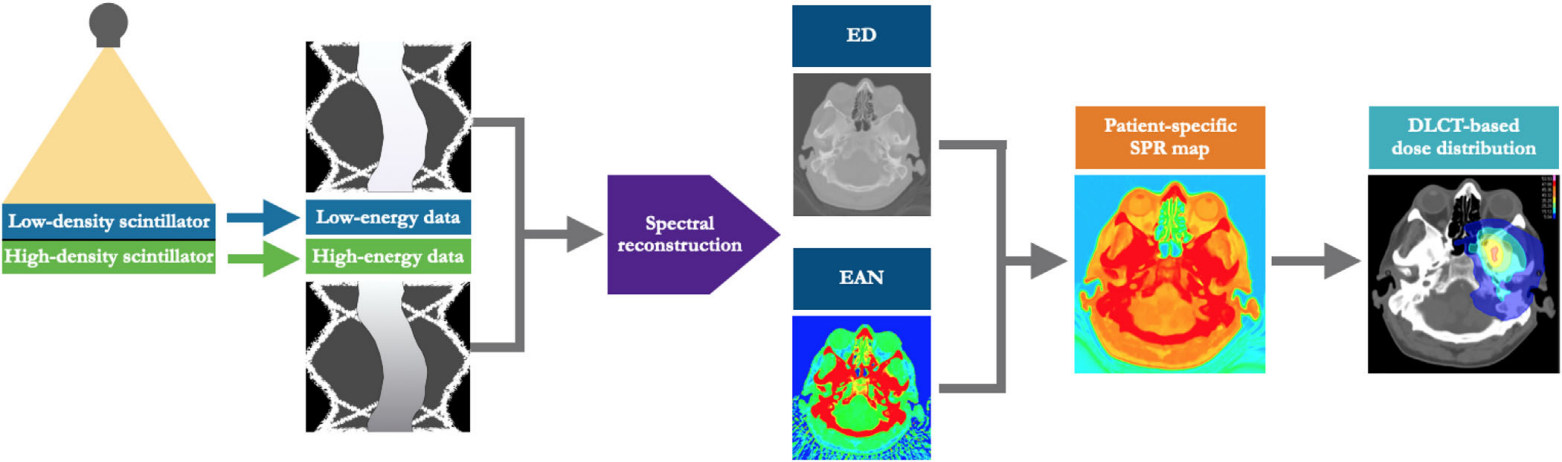
Principle of dual‐layer spectral CT (DLCT)‐based stopping power ratio relative to water (SPR) prediction for particle therapy treatment planning. Simultaneous acquisition of low‐ and high‐energy data, with a detector made of two layers that simultaneously detect two energy levels, allows for projection‐space spectral decomposition. After decomposition, the data are reconstructed and processed to obtain spectral images, i.e., relative electron density (ED) and effective atomic number (EAN), in order to predict SPR and to perform particle therapy treatment planning

To survey the feasibility of performing DLCT‐based treatment planning, this study established a workflow for DLCT‐based particle therapy treatment planning for potential clinical translation. For this purpose, DLCT‐based treatment planning was designed and validated by first deriving 3D maps of SPR, followed by devising a methodology to perform DLCT‐based dose calculation for particle therapy. The entire principle of DLCT data‐based SPR prediction for treatment planning is shown in Figure 1.

For DLCT‐based SPR prediction, the SPR was approximated with the Bethe formula, neglecting higher order correction terms.^10^ Because SPR exhibits a minimal energy dependence in the therapeutic range,^24^ a fixed kinetic energy of 100 MeV for all particle beams was assumed for SPR prediction. The approximation of a fixed value was based on previous work recommending an “effective energy” in SPR estimation of 100 MeV, whereby the uncertainties in energy dependence could be best compensated for clinical applications.^25^ A mean excitation energy (*I*‐value) for water of 78.73 eV was assigned,^26^ consistent with previously reported results ((78 ± 2) eV).^27^ The *I*‐value of the tissue was approximated using a widely referenced parametric method converting EAN in *I*‐value maps.^28^ The exponent to derive EAN from the material‐specific elemental composition weighted by the fraction of electrons associated with each element was 2.94, Philips’ choice which is in correspondence to the Mayneord formula^29^ and other publications.^21^ For each DLCT image acquisition, processing of the raw spectral base image output yields 3D maps of ED and EAN, which in turn are used for SPR computation.^20^ 3D maps of SPR were generated via an in‐house software that takes ED and EAN images and produces a corresponding SPR map that can be read by our clinically employed TPS. Up to now, the commercial TPS at our facility does not allow treatment planning based on SPR maps. However, this study established a workaround for implementing treatment planning based on DLCT‐based SPR images with protons, helium, and carbon ions. For this purpose, we implemented an one‐to‐one conversion curve in the current CT number‐to‐SPR conversion definition required by the TPS and, subsequently, directly imported SPR images based on DLCT in the TPS.

For SECT‐based SPR prediction, the clinical approach of our facility^30^ based on a two‐parameter stoichiometric method^10^, ^31^ was used to generate a CT protocol‐specific HLUT (depicted in Supplementary Material (SM) S1), which was calibrated based on CT image data of body tissue surrogates (Gammex Electron Density CT Phantom 467, Gammex‐RMI, Middleton, WI, USA) from the adult head scan protocol (cf. section 2.3).

### 2.5 Assessment of DLCT data‐based SPR prediction in head patients

In the first investigation, SPR predictions in homogeneous tissue regions were compared between SECT and DLCT image data sets. For each patient, circular regions‐of‐interest (ROIs) of equal size were placed in five reasonably homogeneous tissue regions, similar to Taasti et al.^13^ (depicted in SM S2). The ROIs were placed at exactly the same position in the SECT and DLCT data sets. The brain was segmented by placing circular ROIs (covering an area of ∼100 mm^2^, ∼640 voxels) in ten image slices in the homogeneous brain region above the level of the lateral ventricles. For the cranial bone in the calvaria, ROIs (of ∼50 mm^2^, ∼320 voxels) in ten slices in the upper part of the head were included in the analysis, from the top of the eyes upward. A circular ROI was placed in each eye (of ∼100 mm^2^, ∼640 voxels) and in each lateral ventricle (of ∼50 mm^2^, ∼320 voxels) in five consecutive slices, respectively. For the skull base bone, ROIs (of ∼25 mm^2^, ∼160 voxels) in ten slices in the inferior part of the skull were included. Altogether, ∼20 800 voxels were analyzed per patient in the SECT and DLCT data sets. Subsequently, the ROIs were evaluated quantitatively in terms of mean SPR using an image analysis software (syngo.via, version VB40A, Siemens Healthcare GmbH, Erlangen, Germany). Statistical analysis of SPR comparison between DLCT‐ and SECT‐based methods is described in detail in SM S3.

### 2.6 Assessment of DLCT data‐based treatment planning in head patients

Following investigations of SECT‐ versus DLCT‐based SPR predictions in homogeneous tissue regions (cf. section 2.5), a comparative patient planning study was performed to assess the performance of DLCT and identify which tumor sites would benefit the most from DLCT‐based treatment planning. The treatment planning study was, wherever possible, conducted according to the recommendations of the Radiotherapy Treatment plannINg study Guidelines (RATING).^32^ Proton treatment planning in six head patients from the patient cohort was evaluated. Two patients were excluded, because one patient wore earrings during image acquisition (evoking streak artifacts) and one patient had a hemicraniectomy that would have complicated treatment planning. For each patient, four different realistic treatment plans were created based on patient cases treated with proton therapy at HIT. Therefore, the number of simulated treatment plans was 24. Helium and carbon ion therapy planning was investigated in one patient (patient #1) to compare the impact of DLCT‐based SPR predictions among different ions. Tumor characteristics (i.e., size, depth, location, etc.) were chosen to cover various clinical cases (astrocytoma, meningioma, oligodendroglioma, and pineal region tumor) with the details given in SM S4. For each investigated indication, a physician selected a clinically representative plan from our institution treated with proton beams to be referenced as a “template” for designing the simulated patients using the diagnostic DLCT‐based images. Plan A was selected to evaluate a hypothetical planning target volume (PTV), with most of its volume situated in the brain, that would be treated with three beams. Plan B was created to cover a smaller hypothetical skull‐based tumor with two nearly opposing beams. Compared with plan A, plan C covered a quite similar treatment volume, but would only be treated with two beams separated by 60°. Plan D was chosen for a centrally located tumor in the brain with two nearly opposing fields. The PTV for each treatment plan was defined, and organs‐at‐risk (OARs) were contoured in the CT images using atlas‐based segmentation.^33^

Treatment planning and optimization using multifield optimization with a dose grid of 0.2 cm were performed with RayStation TPS v10 (RaySearch Laboratories AB, Stockholm, Sweden) with the proton Monte‐Carlo dose engine or with the pencil beam dose engine for helium and carbon ions. A fixed relative biological effectiveness (RBE) of 1.1 for protons was assumed. For helium ion therapy, the modified microdosimetric kinetic model (mMKM) was used.^34^ In carbon ion therapy, the radiobiological local effect model (LEM) was employed.^35^ Although the robust optimization concept is under investigation at HIT, it is not yet the clinical standard. Thus, we decided to use the PTV margin concept for optimization, consistent with our current clinical practice. Treatment planning was performed on the PTV with one extra energy layer in the distal margin, laterally with half a spot spacing. Intracranial OARs were delineated based on guidelines by Scoccianti et al.^36^: right and left eyes, optic chiasma, right and left cochlea, right and left hippocampus, brain, brainstem, pituitary gland, right and left inner ears, right and left mandibular condyles, right and left lens, right and left optic nerves, right and left lacrimal glands. The atlas‐based segmentation was used for all OARs, but for several patients manual editing of some structures was still needed. For optimization, dose‐volume parameters were defined as objectives. In a first step, objectives for the PTV and external contour were chosen: minimum dose to PTV of 95% of the prescribed dose, maximum dose to PTV of 103% of the prescribed dose, uniform dose to PTV of 100% of the prescribed dose, minimum dose of 98% of the prescribed dose to 98% of the volume, and dose fall‐off at the external contour. In a second step, objectives for OARs were added according to the “template” treatment plans, whereby for each treatment plan different OARs were considered using the following optimization functions: dose‐volume histogram (DVH) functions for OARs and Max EUD (equivalent uniform dose) functions, *a* = 1, corresponding to a mean dose constraint. PTV coverage was similar for proton, helium, and carbon ion treatment plans for comparisons between the different ions.

**FIGURE 2:**
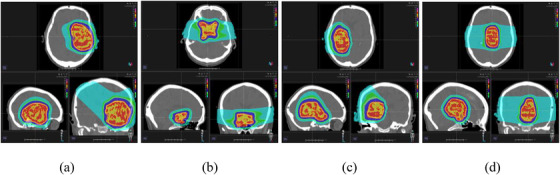
Proton treatment plan design for a study patient (patient #1) with RayStation treatment planning system (TPS) showing (a) plan A (astrocytoma), (b) plan B (meningioma), (c) plan C (oligodendroglioma), and (d) plan D (pineal region tumor)

For each patient, treatment planning was performed on the SECT‐based approach, as depicted in Figure 2. Plan acceptability was decided based on the clinical patient cases that were used as “template” treatment plans. Subsequently, the dose distributions were recalculated on DLCT‐based SPR images using the same beam parameters without reoptimization.

Patient plans calculated with SECT and DLCT were then compared in terms of their range prediction and additional dose calculation features including PTV coverage and evaluation of dose differences to OARs. Differences in range prediction were analyzed with line‐dose profiles in beam direction (using RayStation TPS) and quantified by absolute range shifts at the distal range at 90% (R_90_) and 80% (R_80_) of prescribed dose (∆R_90_  =  | R_90,SECT_ − R_90,DLCT_ | and ∆R_80_  =  | R_80,SECT_ − R_80,DLCT_ |). For all patients and plans, five equidistant line‐dose profiles (ten equidistant line‐dose profiles for proton, helium, and carbon ion treatment plans in patient #1) per beam were evaluated inside each PTV (cf. Figure 5a). Relative range differences were calculated by dividing ∆R_90_ (∆R_80_) by R_90,DLCT_ (R_80,DLCT_):

(1) $$\;\frac{\Delta R_{90}}{R_{90,\mathrm{DLCT}}}=\frac{\left|R_{90,\mathrm{SECT}}-R_{90,\mathrm{DLCT}}\right|}{R_{90,\mathrm{DLCT}}}$$

(2) $$\frac{\Delta R_{80}}{R_{80,\mathrm{DLCT}}}=\frac{\left|R_{80,\mathrm{SECT}}-R_{80,\mathrm{DLCT}}\right|}{R_{80,\mathrm{DLCT}}}$$

To determine whether DLCT imaging had a significant effect on range prediction, a *t* test for two paired samples with a significance level of 5% was applied. In addition, the intrapatient (within a patient) and interpatient (between patients) variabilities of range shifts were calculated, which were defined in previous work as mean of the standard deviation and as standard deviation of the mean of patient‐specific range shifts, respectively.^14^ Dose distributions were compared using a 3D gamma analysis^37^ for local calculation with a passing criterion of 1%/1 mm using a low dose cutoff of 5% of the maximum dose. Additionally, DVHs were compared in terms of absolute dose differences in the mean or maximum dose over the entire treatment (total dose) for each OAR, respectively. The target coverage was assessed by the PTV D_99%_ dose. The PTV is more sensitive to range shifts compared with the CTV, because changes in range directly impact the PTV coverage, but not necessarily the CTV.

## 3 RESULTS

### 3.1 Evaluation of DLCT data‐based SPR prediction in head patients

**FIGURE 3:**
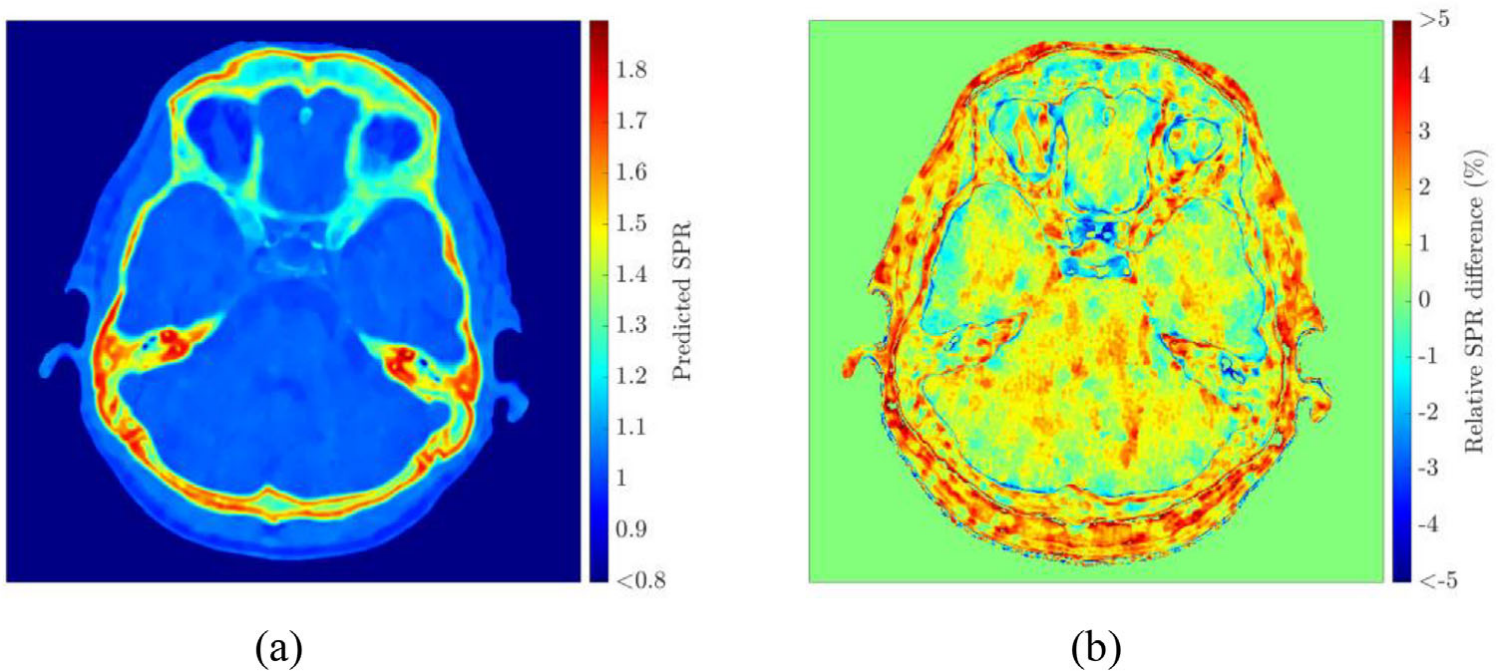
Axial plane of patient #1 showing predicted stopping power ratio (SPR) with dual‐layer spectral CT (DLCT) images (a) and relative difference between SPR derived using DLCT and single‐energy CT (SECT) (b)

Feasibility and accuracy of the DLCT‐based SPR prediction were first investigated in homogeneous anatomical regions in a patient cohort. In Figure 3, the SPR prediction and relative differences between SPR maps derived using DLCT and SECT are plotted for patient #1. The largest SPR differences between DLCT and SECT were found in air‐filled cavities and bone tissue, whereby the SPR differences were negative for air‐filled cavities and positive for bones.

**FIGURE 4:**
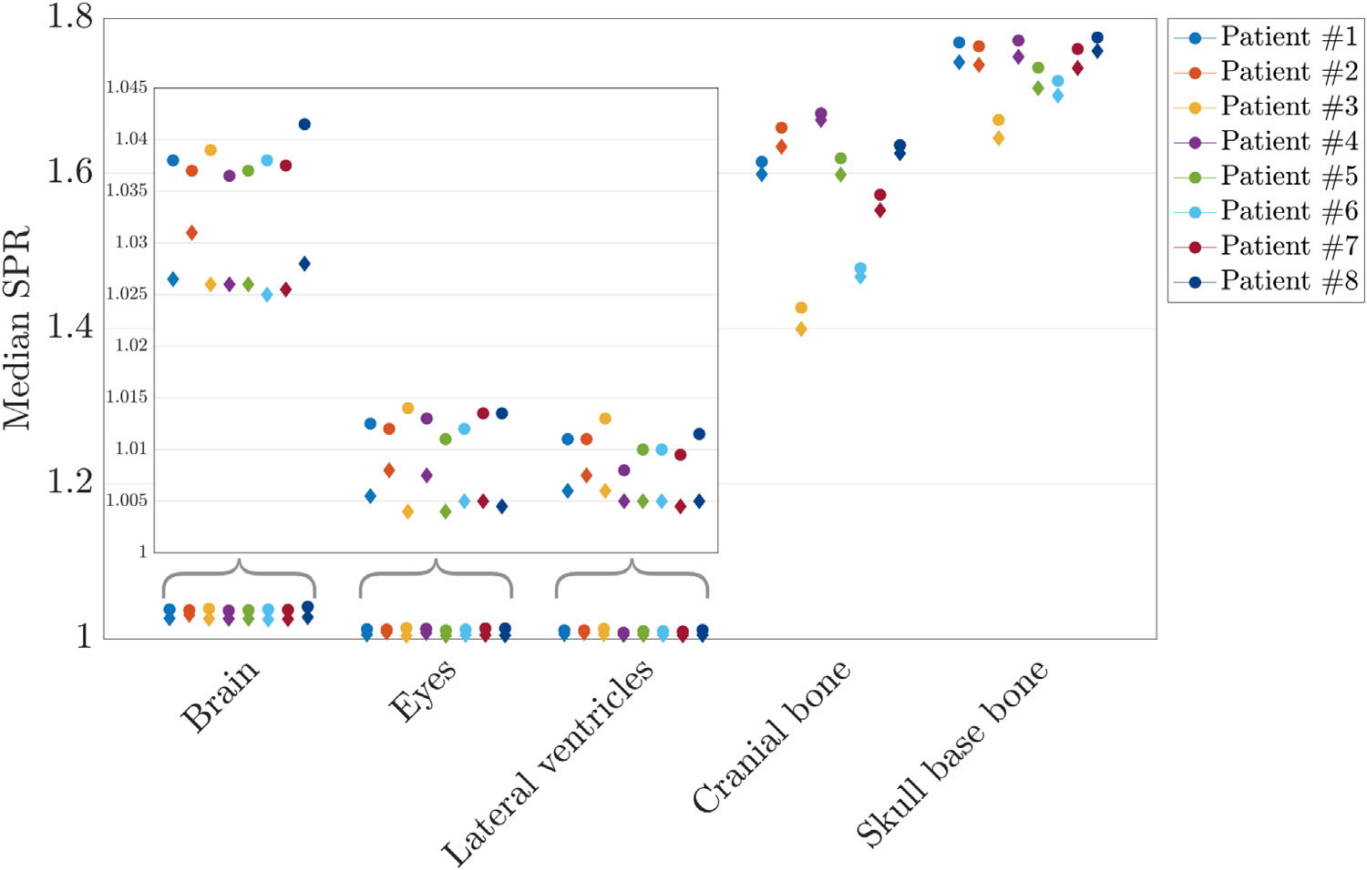
Median stopping power ratio (SPR) values predicted with dual‐layer spectral CT (DLCT) (marked with circles) and single‐energy CT (SECT) (marked with diamonds) over all investigated slices for all investigated regions‐of‐interest (ROIs) in each patient showing brain, eyes, lateral ventricles, cranial bone, and skull base bone. The subplot zooms in on the relevant SPR value region for brain, eyes, and lateral ventricles

Quantitative analysis in specific tissue regions was performed per ROI for DLCT‐ and SECT‐based SPR measurement. Figure 4 shows the median SPR value distributions for DLCT and SECT over all patients for the five ROIs. For all patients, mean SPR differences over five defined ROIs were positive, showing higher SPR estimates based on DLCT than on SECT (Table 1). The mean SPR difference was (1.10 ± 0.07)% in brain, (1.13 ± 0.17)% in cranial bone in the calvaria, (0.69 ± 0.06)% in eyes, (0.48 ± 0.05)% in lateral ventricles, and (1.22 ± 0.14)% in skull base bone. The percentage difference ranged from 0.32% to 1.87% over all ROIs and was 0.87% in the median (cf. Table 1). The standard error of the mean in bony structures was higher than in brain, eyes, and lateral ventricles.

**TABLE 1:** Stopping power ratio (SPR) difference for regions‐of‐interest (ROIs) in brain, cranial bone, eyes, lateral ventricles, and skull base bone

	SPR difference${\bar{\delta }}_{\mathrm{SPR}}\pm \left(\delta_{SPR}\right)\left(\%\right)$				
Patient #	Brain	Cranial bone	Eyes	Lateral ventricles	Skull base bone
1	1.14 ± 0.09	1.28 ± 0.47	0.70 ± 0.13	0.47 ± 0.12	1.46 ± 0.54
2	0.68 ± 0.17	1.39 ± 0.16	0.38 ± 0.08	0.33 ± 0.08	1.14 ± 0.51
3	1.28 ± 0.13	1.87 ± 0.67	0.92 ± 0.05	0.64 ± 0.05	1.10 ± 0.47
4	1.02 ± 0.28	0.65 ± 0.24	0.50 ± 0.16	0.32 ± 0.14	1.24 ± 0.38
5	1.08 ± 0.06	0.96 ± 0.27	0.72 ± 0.11	0.57 ± 0.17	1.35 ± 0.37
6	1.21 ± 0.08	0.76 ± 0.30	0.64 ± 0.10	0.42 ± 0.17	1.08 ± 0.28
7	1.14 ± 0.16	1.49 ± 0.21	0.75 ± 0.16	0.50 ± 0.10	1.46 ± 0.32
8	1.22 ± 0.11	0.66 ± 0.09	0.89 ± 0.08	0.60 ± 0.12	0.92 ± 0.41
Median	1.16	1.07	0.69	0.50	1.25
Mean ± SEM	1.10 ± 0.07	1.13 ± 0.17	0.69 ± 0.06	0.48 ± 0.05	1.22 ± 0.14

*Note*: The arithmetic mean of the relative SPR difference $({\bar{\delta }}_{\mathrm{SPR}})$ is given along with the standard deviation (s(δ_SPR_)) for each patient. Median and mean along with the standard error of the mean (SEM) over each ROI are indicated.

The performed *t* test rejected the null hypothesis at the 5% significance level, i.e., difference of mean SPR values for SECT and DLCT was nonzero. The SPR prediction based on DLCT was significantly different (*p* < 0.05) from the SPR prediction based on SECT. The mean relative difference in SPR prediction (δ_SPR_) over the ROIs was 0.92%, with a standard error of the mean of 0.45%. The 95% confidence interval for SPR shifts was [0.88, 0.97]%.

### 3.2 Evaluation of DLCT data‐based treatment planning in head patients

**FIGURE 5:**
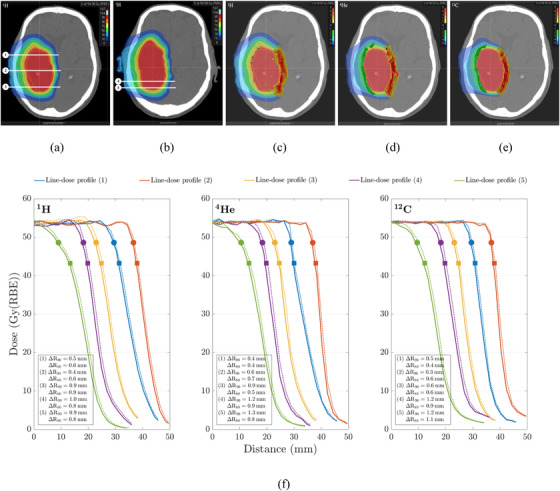
For a representative patient case, proton therapy dose distribution of plan C for patient #1 in two different axial slices (a, b), dose difference map superimposed on the dose distribution shown in (a) for protons (c), for helium ions (d), for carbon ions (e), and five representative line‐dose profiles calculated on dual‐layer spectral CT (DLCT) (solid line) and single‐energy CT (SECT) (dotted line) for protons, helium, and carbon ions to quantify deviations in range prediction (f). The placement of the five line‐dose profiles in (f) are illustrated in (a, b). The illustrated depth‐dose curves indicate absolute range (R) differences between DLCT and SECT at R_90_ (marked with circles) and R_80_ (marked with squares)

**FIGURE 6:**
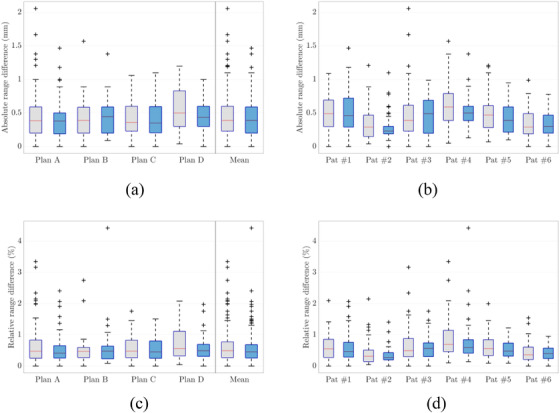
(a, b) Box plots showing deviations in beam's eye view (BEV) range (R) prediction between single‐energy CT (SECT)‐ and dual‐layer spectral CT (DLCT)‐based proton treatment planning (∆R  =  | R_SECT_ − R_DLCT_ |). (c, d) Box plots showing relative differences in range prediction ($\frac{\Delta R}{R_{\mathrm{DLCT}}}$) (cf. equations 1 and 2). On each box, the central mark (red) indicates the median, and the bottom and top edges of the box indicate the 25th and 75th percentiles, respectively. The whiskers extend to the most extreme data points (i.e., smallest observation ≥ lower quartile − 1.5 × interquartile range/largest observation ≤ upper quartile + 1.5 × interquartile range), and the outliers are plotted individually using the + symbol. In gray are the results depicted for R_90_, and in blue are the results shown for R_80_. (a, c) Analysis for each plan and (b, d) analysis for each patient (Pat)

Figure 5 shows exemplary proton therapy dose distributions and dose difference maps of patient #1 for protons, helium, and carbon ions as well as the corresponding line‐dose profiles for DLCT‐ and SECT‐based calculation of the depicted slices. Absolute and relative range shifts at 90% and 80% dose fall‐off in proton treatment plans between the two SPR predictions are summarized in Figure 6. The plots in Figure 6 depict the results for (i) each of the four plans combining the data for the six patients and (ii) each patient combining the data for the four plans using box plots to visualize the beam's eye view (BEV) range differences. For all patients, there was a statistically significant (*p* < 0.05) difference between the range predicted by SECT and DLCT. The shift of absolute (relative) range differences between SECT and DLCT lay in the interval [0.42, 0.47] mm ([0.54, 0.62]%) with a probability of 95%. Mean absolute range shift over 270 evaluated line‐dose profiles in the virtual brain tumors between DLCT and SECT was (0.46 ± 0.32) mm at R_90_ and (0.42 ± 0.26) mm at R_80_, with a maximum absolute range difference of 2.06 mm at R_90_ and of 1.47 mm at R_80_. The range shift over both evaluated dose fall‐off points was (0.44 ± 0.29) mm in the mean and with a median of 0.39 mm. The 25th percentile of the distribution was calculated to be 0.20 mm, and the 75th percentile of the distribution to be 0.59 mm. The relative differences are summarized in Table 2. The median relative range difference was 0.6% over all investigated treatment plans. Moreover, the intrapatient variability (cf. Figure 6d) of relative range shifts with a value of 0.44% was larger than the interpatient variability (cf. Figure 6c) of 0.07%. The differences between intrapatient and interpatient variability are in part caused by considerably large differences between the chosen hypothetical treatment plans and, thus, differences in the traversed tissues in terms of tissue type and amount.

**TABLE 2:** Relative proton range differences given in percent ($\frac{\Delta R}{R_{\mathrm{DLCT}}}$) (cf. Equations 1 and 2)

		Relative range differences (%)				
		Mean	25th Percentile	50th Percentile (median)	75th Percentile	100th Percentile
Plan A	R_90_	0.66	0.25	0.48	0.83	3.35
	R_80_	0.52	0.26	0.41	0.65	2.41
Plan B	R_90_	0.50	0.27	0.47	0.60	2.75
	R_80_	0.54	0.23	0.48	0.64	4.43
Plan C	R_90_	0.58	0.27	0.48	0.83	1.76
	R_80_	0.57	0.25	0.45	0.80	1.51
Plan D	R_90_	0.70	0.33	0.56	1.11	2.08
	R_80_	0.57	0.31	0.49	0.69	1.98
All plans	R_90_	0.61	0.27	0.49	0.78	3.35
	R_80_	0.55	0.26	0.46	0.68	4.43

**FIGURE 7:**
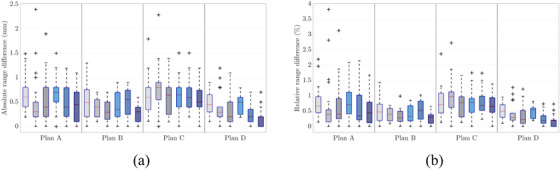
(a) Box plot showing deviations in beam's eye view (BEV) range (R) prediction for the three ions (^1^H, ^4^He, ^12^C) between single‐energy CT (SECT)‐ and dual‐layer spectral CT (DLCT)‐based treatment planning (∆R  =  | R_SECT_ − R_DLCT_ |). (b) Box plots showing relative differences in range prediction ($\frac{\Delta R}{R_{\mathrm{DLCT}}}$) (cf. equations 1 and 2). On each box, the central mark (red) indicates the median, and the bottom and top edges of the box indicate the 25th and 75th percentiles, respectively. The whiskers extend to the most extreme data points (i.e., smallest observation ≥ lower quartile − 1.5 × interquartile range/largest observation ≤ upper quartile + 1.5 × interquartile range), and the outliers are plotted individually using the + symbol. The plots show the analysis for each plan in patient #1. For each individual plan, the plots depict the results for ^1^H (left), ^4^He (middle), and ^12^C (right). In gray shades are the results depicted for R_90_, and in blue shades are the results shown for R_80_

Figure 7 shows the absolute and relative range shifts for the four plans observed in patient #1 for the three ions (^1^H, ^4^He, and ^12^C). The absolute range shift over both evaluated dose fall‐off points was (0.58 ± 0.16) mm (^1^H), (0.49 ± 0.19) mm (^4^He), and (0.41 ± 0.17) mm (^12^C) in the mean, and with a median of 0.60 mm (^1^H), 0.40 mm (^4^He), and 0.31 mm (^12^C). The helium and carbon ion range shifts between SECT and DLCT were in line with those of protons, even though there are R_90_ or R_80_ variations among the three particles in the individual plans.

The general agreement between DLCT‐ and SECT‐based dose calculations was confirmed in the evaluation of the clinical patient treatment plans. 3D gamma analysis of the dose distributions revealed good agreement between DLCT‐ and SECT‐based treatment planning with a mean 3D gamma local pass rate (1%/1 mm) of 97.3% over all patients and treatment plans, ranging from 96.4% (plan A) to 96.7% (plan D) to 97.7% (plan C) and 98.3% (plan B). Despite good agreement in 3D gamma analysis, there were differences between DLCT and SECT with regard to PTV coverage and dose to OARs.

**FIGURE 8:**
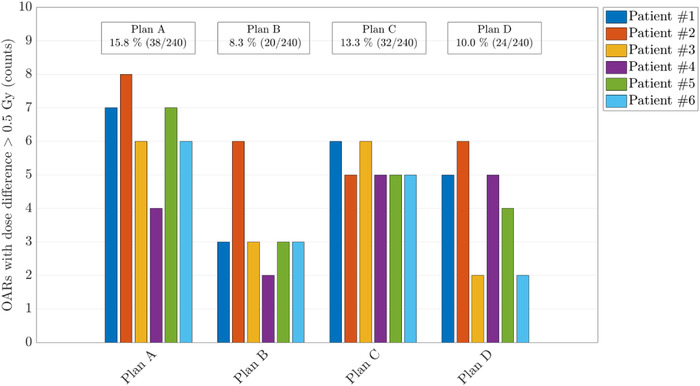
Dose differences in the mean or maximum dose of more than 0.5 Gy (RBE) in the total plan of all evaluated OARs (considering only proton treatment planning). Each color represents one of the six evaluated patients

**FIGURE 9:**
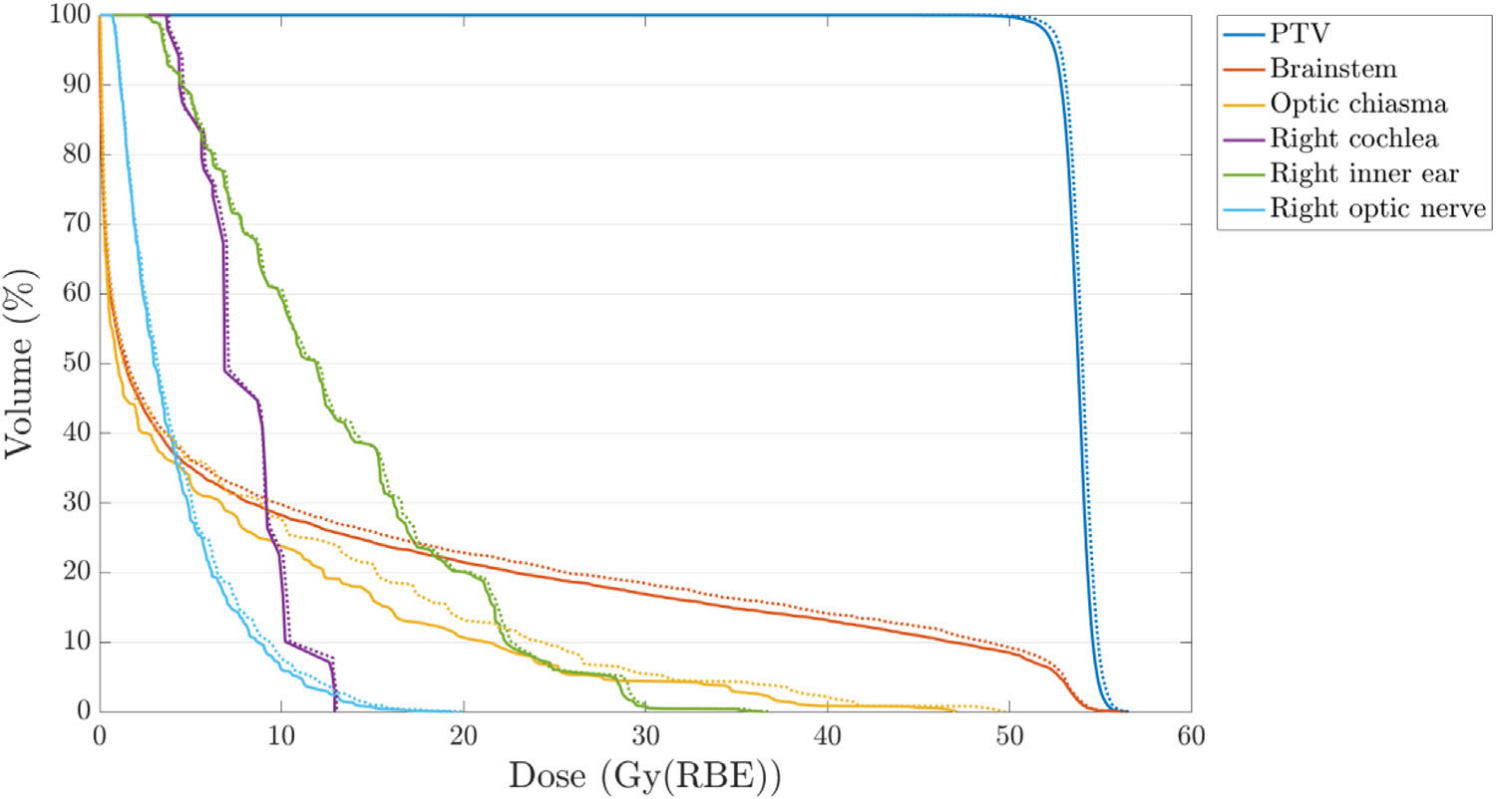
Representative DVH for patient #1, treatment plan C using protons, calculated on dual‐layer spectral CT (DLCT) (solid line) and single‐energy CT (SECT) (dotted line) data sets displaying all structures used for optimization

In Figure 8, relevant dose differences are shown for all evaluated OARs in each patient and plan, respectively. In 12% of all evaluated OARs, the results indicated differences in the mean or maximum (D_0.03cc_) dose of more than 0.5 Gy (RBE) and differences up to 6.8 Gy (RBE) in the total plan. The average (and maximum) criterion was reached 46 (and 68) times over all patients and proton plans. DLCT‐based recalculation of the SECT‐optimized treatment plans showed a decrease in PTV coverage, as evaluated with the difference in PTV D_99%_, of 1.0% or 0.5 Gy (RBE) in the mean over all evaluated plans and patients (Table 3). With regard to the three ions, Table 3 indicates quite similar differences for ^4^He and ^12^C in PTV coverage between SECT and DLCT compared with ^1^H. Figure 9 shows the DVH of plan C for an example patient (patient #1). In the optic chiasma (located close to the target dose fall‐off), the maximum dose (D_0.03cc_) was 49.71 Gy (RBE) for SECT‐based and 47.06 Gy (RBE) for DLCT‐based treatment planning, a decrease of 6%. A higher SPR value (as seen in bony structures and brain in Figure 4) leads to a shorter range, which resulted in a dose decrease in the optic chiasma in the given situation. The optic chiasma is a serial structure in which disabling any subunit causes the entire organ to fail.^38^ In patient #1, differences in the mean or maximum dose of more than 0.5 Gy (RBE) over all evaluated OARs were observed in 13% for ^1^H, in 9% for ^4^He, and in 6% for ^12^C. In the DVHs, the dose to distal OARs decreased using ^4^He or ^12^C, due to the sharper gradients of helium and carbon ions compared with protons (cf. Figure 5f). Therefore, the absolute dose differences to OARs between SECT and DLCT were also smaller compared with protons. Nevertheless, with a longer range using DLCT and sharper gradients, the dose there could be more than in the SECT plan; therefore, it is very patient‐specific.

**TABLE 3:** Differences in planning target volume (PTV) coverage between single‐energy CT (SECT) and dual‐layer spectral CT (DLCT)

	PTV coverage difference ∆D_99%_ (Gy (RBE))			
Patient #	Plan A	Plan B	Plan C	Plan D
1 (^1^H)	0.51	0.78	0.61	0.74
1 (^4^He)	0.17	0.36	0.38	0.35
1 (^12^C)	0.36	0.50	0.47	0.42
2	0.07	0.30	0.34	1.16
3	0.34	0.86	0.41	0.47
4	0.46	0.36	0.72	0.84
5	0.37	0.45	0.79	0.58
6	0.01	0.11	0.43	0.43
$\bar{\Delta D}$_99%_ ± s(∆D_99%_))	0.29 ± 0.21	0.48 ± 0.29	0.55 ± 0.18	0.70 ± 0.27

*Note*: Differences in PTV coverage between SECT‐ and DLCT‐based dose calculations (∆D_99%_  =  D_99%,SECT_ − D_99%,DLCT_) for each treatment plan and patient. The arithmetic mean ($\bar{\Delta D}$_99%_) in PTV coverage is indicated along with the standard deviation (s(∆D_99%_)) for each plan (only proton treatment planning is included in the calculation).

## 4 DISCUSSION

This study evaluated the clinical relevance of DLCT‐based SPR prediction for proton, helium, and carbon ion beam therapy treatment planning in the brain. A comprehensive workflow for DLCT‐based ion beam therapy treatment planning was established (cf. section 2.4). Through a patient cohort study in homogeneous tissue regions and heterogeneous patient scenarios, DLCT‐ and SECT‐based SPR differences and their dosimetric impact were investigated and compared. The clinical viability of DLCT‐based SPR prediction and its feasibility for performing particle therapy treatment planning were assessed to justify its clinical use. Depending on the anatomical regions, SECT‐ and DLCT‐based methods produced variant degrees of SPR prediction differences in the studied patient cohort. Owing to the results from preclinical studies showing better SPR prediction with DLCT compared with SECT^18^, ^20^, ^21^, ^22^, ^23^ and the observed differences in SPR prediction in this study, DLCT may be justifiably better for clinical practice in patient treatments where the beams intercept and traverse heterogeneous anatomical regions.

First, SPR differences in various homogeneous tissue regions were analyzed on a per‐patient basis, and the determinants leading to the largest uncertainties were identified and quantified. The relative SPR comparison in the patient cohort showed statistically significant SPR differences in all investigated anatomical regions between DLCT‐ and SECT‐based methods. Furthermore, Table 1 shows the interpatient variability of SPR predictions. Bony tissues showed the largest deviation between DLCT and SECT of the investigated ROIs, potentially due to their high SPR values. The SPR differences of 1.1% to 1.2% seen in bone (cf. Table 1) could potentially imply a benefit in DLCT‐based treatment planning, assuming a more accurate DLCT‐based SPR prediction in patient anatomies. The SPR differences of about 1.1% in the brain, which is often the main tissue type in the beam, could also be of clinical relevance. For instance, SPR differences of 1%, i.e., translating into range differences of 1%, result in 1 mm range shift over 10 cm depth in the body. Beyond that, DLCT could be advantageous for tumors nearby critical OARs like the optical system or brainstem. Although the SPR differences were above 1% for ROIs uniformly composed of bony tissue or brain, median relative range deviations in the patient as a whole were 0.6%. The difference can be attributed to the dependence on anatomical target site and composition in treatment planning and the compounding effects of SPR prediction power of various heterogeneous tissues composed of bone and soft tissue.

Second, absolute and relative range differences and the dosimetric impact of DLCT‐based SPR calculation in comparison with the SECT‐based approach were carefully assessed. The influence of SPR uncertainty on patient dose uncertainty is not trivial and substantially case‐dependent. Comparison of DLCT‐based proton treatment plans of four brain tumor locations to the corresponding SECT plans showed considerable differences in SPR at voxel level and a mean relative range difference of about 0.6% at the distal fall‐off were observed (cf. Table 2); in certain cases, the range shift might be of clinical relevance. The DVHs showed a decrease in the mean and maximum OAR dose using DLCT owing to the SPR difference between DLCT and SECT. The 25th and 75th percentiles varied from 0.23% to 1.11% across the six patients. Range shifts and dose differences in OARs between DLCT and SECT in helium and carbon ion treatment plans were similar to those of protons (cf. Figure 7). Despite the intrapatient and interpatient variability, the example cases showed clinically relevant range differences between SECT‐ and DLCT‐based SPR predictions. Furthermore, the large intrapatient variation of range shifts illustrates that variation in range uncertainty depends on the anatomical structure and the beam path. In turn, the magnitude of improvement in range prediction with DLCT depends on the treatment location and its heterogeneity.

Similar studies have previously been performed comparing DECT‐ and SECT‐based SPR estimation for patients with head tumors. The outcome in SPR prediction differences in homogeneous tissue regions is in line with a study of Taasti et al.,^13^ who investigated ROIs in the cranium, brain, and eyes. The results found in this study are also of the same order as recent studies using other DECT acquisition methods (e.g., consecutive scanning) or other DECT SPR prediction methods, showing that range differences of around 1 mm (1%) may be expected for the brain region.^13^, ^14^ The results from this work are likewise comparable with a study analyzing range shifts obtained in five head trauma patients with simulated base of skull tumors,^39^ reporting median relative range differences of about 0.5%–1%. The median differences found in this study on DLCT‐based range differences are similar to or slightly smaller than those observed in previous studies using other DECT acquisition methods; however, there exist rather high interpatient variabilities as well as larger differences for some patients. One should also take into consideration that the SECT‐based prediction method applied in this study, using an HLUT divided into ten line segments, may be more methodologically demanding in the context of HLUT generation than in previous studies, that used an HLUT divided into three parts representing different tissue types along with different slopes of the respective line segments. A higher number of HLUT line segments might be already better suited for SPR prediction. A recent survey‐based study revealed a large intercenter variability in HLUT definition, showing that the number of HLUT line segments varied widely between 2 and 11.^40^ Hence, the applied HLUT in this study is at the upper end of the line segment number spectrum. In the context of range differences between DLCT and SECT observed in this study specifically, one must note that HIT implements highly refined treatment planning protocols that have evolved since facility start‐up in 2009. Consequently, the facility has gained valuable experience in minimizing range uncertainty with the applied HLUT approach and demonstrated that such techniques can provide fairly accurate SPR estimation in controlled treatment scenarios. Nonetheless, benchmarking and comparison within the European Particle Therapy Network regarding CT calibrations using a standardized phantom showed large differences and intercenter variations in range reaching up to 2.9%.^41^ Thus, direct DLCT‐based SPR prediction could lead to reduced differences between centers or help new proton centers begin treatment with a greater confidence in range prediction.

As shown in the hypothetical treatment planning cases, even small discrepancies in the calculated SPR can result in significant changes in range, because they may accumulate over the entire beam path.^6^ Thus, DLCT may lead to clinically relevant range shifts and subsequently dose differences, especially for tumors in challenging locations, e.g., tumors centrally located in the head, deep‐seated, or treated with ion beams traversing a high amount of bony tissue. In turn, the range differences could enable reduced dose to normal tissue and OARs with benefits in PTV coverage (i.e., D_99%_ dose). In particular, this study found differences in the mean or maximum dose of more than 0.5 Gy (RBE) in the total plan (cf. Figure 8) and mean differences in D_99%_ target dose of 0.5 Gy (RBE) (cf. Table 3). Variation in CTV coverage, however, might be even smaller and not clinically relevant. The current conservative safety margins and plan robustness may be reduced if the SPR can be calculated with greater confidence. Even if the observed range differences are below 1 mm in the median, there may be clinically significant differences for individual patients, as reported in the large intra‐ and interpatient variability (cf. Figure 6), which may be highly relevant for increasing personalized medicine considerations.^32^ Recent work demonstrates the benefits in terms of normal tissue complication probability (NTCP) in mitigating range uncertainty even for smaller reductions.^42^ The study showed that higher range differences might be expected for beams traversing heterogeneous tissues with SPR values that differ considerably compared with the SPR of water (e.g., bone tissue, air‐filled cavities) (cf. Figure 4 and Table 1). Thereby, the accuracy of SPR in each voxel in the patient determines the accuracy of the range calculation. More different tissue types in the beam path can lead to larger deviations in range prediction (as already observed in previous studies^14^). Therefore, a patient‐specific DLCT‐based SPR prediction with high accuracy in each individual tissue type would be advisable. In particular, DLCT may be beneficial in complex cases; however, as of now it is difficult to identify in advance which patients would most benefit from DLCT‐based treatment planning, and so the use of DLCT may be advisable for all patients. DLCT‐based SPR calculation may even raise the possibility of using contrast agent during planning CT image acquisition^43^ and may be beneficial in the presence of metal implants, surgical stabilizations, or other special materials (e.g., liquid embolic agents), or in the presence of image artifacts (e.g., produced by metal implants).^4^

To judge which of the two evaluated approaches is closer to reality, the respective SPR accuracy must be known.^14^ For instance, precise range verification with prompt gamma imaging^44^, ^45^, ^46^ or proton transmission imaging^47^, ^48^ could provide millimeter accuracy in range verification, but in its current state is not clinically widespread.^49^ Thus, the accuracy of DLCT‐based SPR prediction in patients has yet to be verified directly. Instead, SPR accuracy was demonstrated indirectly by translating the results shown in previous studies^18^, ^20^, ^22^, ^23^ to patient treatment planning. In tissue substitutes, predicted SPR values were within a mean accuracy of 0.6% compared with measured SPR and showed substantially better agreement with measured data compared with standard CT‐number‐to‐SPR calibration with a mean deviation of 1.5%.^20^ Beyond that, SPR prediction with DLCT outperformed the clinical SECT standard in a half‐head anthropomorphic phantom with a range prediction improvement of 1 mm,^20^ when using a single beam directed through highly heterogeneous structures. A similar study acquired ground‐truth measurements in an anthropomorphic head phantom showing better agreement between DECT and measured SPR compared with SECT.^50^ The current study used two or three beam directions, directed through heterogeneous as well as relatively homogeneous tissue regions (e.g., brain). The order of magnitude of SPR prediction difference between DLCT and SECT in phantoms was similar to the examined patient cases in this study. Ideally, in this study, using a patient cohort, a ground‐truth measurement for SPR would be referenced. However, this study aimed to evaluate whether clinically relevant SPR and therefore range deviations occurred between SECT and DLCT in a patient cohort, justifying whether more sophisticated image acquisition tools would be beneficial and may be considered for potential clinical implementation. As DLCT has been shown to be superior to SECT in tissue surrogates and an anthropomorphic phantom, the dissimilar results for DLCT and SECT observed in this patient study could imply that DLCT would improve the dose accuracy in ion beam therapy treatment planning.

In this study, the feasibility of direct patient‐specific SPR prediction based on DLCT could be demonstrated using the existing clinical framework and equipment. Compared with other DECT techniques, DLCT imaging using a single X‐ray source is not influenced by patient motion occurring within the time span of acquisition (e.g., breathing, swallowing, organ movements). At the same acquisition dose as conventional CT imaging, DLCT affords a comprehensive spectral data set for each patient, without the need for additional scans or deviations from the clinical protocols. Nevertheless, DLCT imaging has a limited spectral separation between the low‐ and high‐energy data sets because the technique uses a single X‐ray source.^15^ Moreover, cross‐scatter radiation between detector layers can occur.^17^ Additionally, as a result of using the same tube current in both cases, noise level may differ between low‐ and high‐energy images.^15^ A discussion of uncertainties within the study can be found in SM S5.

Further studies may evaluate other anatomical sites (e.g., head and neck tumors) and beams traversing several tissue types and thicker bony structures (e.g., tumors in the pelvic region) as well as beams passing through the lungs (e.g., Hodgkin lymphoma). In brain tumor cases, ion beams penetrate mainly soft tissue. Within treatment fields of prostate cancer patients, we would expect substantially larger differences, as already observed by Wohlfahrt et al.^14^ Although CT uncertainty can be incorporated into planning robustness optimization, this study followed the current clinical practice at HIT and applied the PTV margin concept. In additional studies, robust optimization might be conducted and compared with regard to dose differences in CTV and OARs, in order to assess the influence of robustly optimized treatment plans in combination with DLCT‐based SPR prediction. Moreover, investigations of patients with real tumors in the brain and range measured in biological tissue samples are essential to confirm the clinical viability of DLCT‐based range prediction. In particular, there exists a large intra‐ and interpatient variation of SPR shifts seen in this radiological patient cohort, which might also cause smaller or bigger range differences in other patient cases and should be further investigated in larger patient cohorts. Further studies with radio‐oncological patient data and “real” clinical indications are important to carry out in order to show that the results may be generalizable and transferable to clinical routine. In spite of this, potential CT artifacts can also affect the accuracy of ion beam range prediction based on CT images, which are particularly severe in the presence of metallic implants.^6^ Hence, the benefit of DLCT can be especially large in the case of nontissue materials such as implants or contrast agent, which in general are not appropriately covered by any conventional CT‐number‐to‐SPR conversion.^19^ Investigations of SPR precision for nontissue samples are foreseen. DLCT imaging may not only improve range prediction, but DLCT data sets could also help in characterizing the implant in terms of ED and EAN. Additional applications of DLCT in both photon radiotherapy and particle therapy are conceivable with more practical benefits, such as simplifying treatment planning workflow, reducing CT simulation time and radiation exposure as well as the anesthesia time for pediatric patients by performing dose calculation on postcontrast DLCT images.^43^ Finally, evaluation of DLCT‐based treatment planning in more patient cases for carbon and helium ion beam therapy is anticipated.

## 5 CONCLUSIONS

This study performed the first analysis of DLCT‐based SPR prediction in the brain. In homogeneous tissue regions, analysis suggests significant mean SPR differences between the DLCT‐based and conventional SECT‐based approaches of about 1%. In heterogeneous anatomical regions, mean proton range shifts in treatment plans between DLCT and the clinical standard of 0.6% were observed, with variations exceeding 4% of the total range. Range shifts between DLCT and SECT in helium and carbon ion treatment plans were similar to those of protons. In particular, DLCT is most advantageous in treatment plans where beams are traversing highly heterogeneous structures. Therefore, patient‐specific DLCT‐based SPR prediction may improve proton, helium, and carbon ion range calculation and eventually lead to reduced range uncertainty margins. In sum, the study demonstrated the feasibility of using DLCT imaging for proton, helium, and carbon ion beam therapy treatment planning and its ability to provide patient‐specific SPR prediction. Further clinical investigations using larger patient cohorts and examining other treatment regions will continue to focus on the inter‐ and intrapatient variability to realistically quantify the possible benefit of DLCT, and consequently to estimate the potential range uncertainty reduction resulting in smaller therapeutic margins for high‐precision ion beam therapy.

## AUTHOR CONTRIBUTIONS

Conceptualization, F.L., W.S., and A.M.; methodology, F.L., T.T., S.H., W.S., and A.M.; data acquisition and analysis, F.L., T.T., and A.M.; interpretation, F.L., T.T., S.M., W.S., and A.M.; writing—original draft preparation, F.L., T.T., and S.M.; writing—review and editing, F.L., T.T., S.M., S.H., J.D., W.S., and A.M.; supervision, J.D., W.S., and A.M.

## CONFLICT OF INTEREST

The authors have no relevant conflicts of interest to disclose. W.S. is a member of the CT Advisory Board of Philips Medical Systems.

## Supporting information

Supporting information
