# Supplementary material for: Dual‐layer spectral CT for proton, helium, and carbon ion beam therapy planning of brain tumors
Source: J Appl Clin Med Phys. 2021 Nov 1;23(1):e13465. doi: 10.1002/acm2.13465 (PMC8803296; doi:10.1002/acm2.13465)
Supplement: Supplementary file 1 — Supporting information [file ACM2-23-e13465-s001.pdf]

Dual-layer spectral CT for proton, helium and carbon ion-beam therapy planning of brain tumors

SUPPLEMENTARY MATERIAL

S1 Single-energy CT-based Hounsfield look-up table

The Hounsfield look-up table (HLUT) derived in the single-energy CT (SECT)-based approach is shown in figure 1.

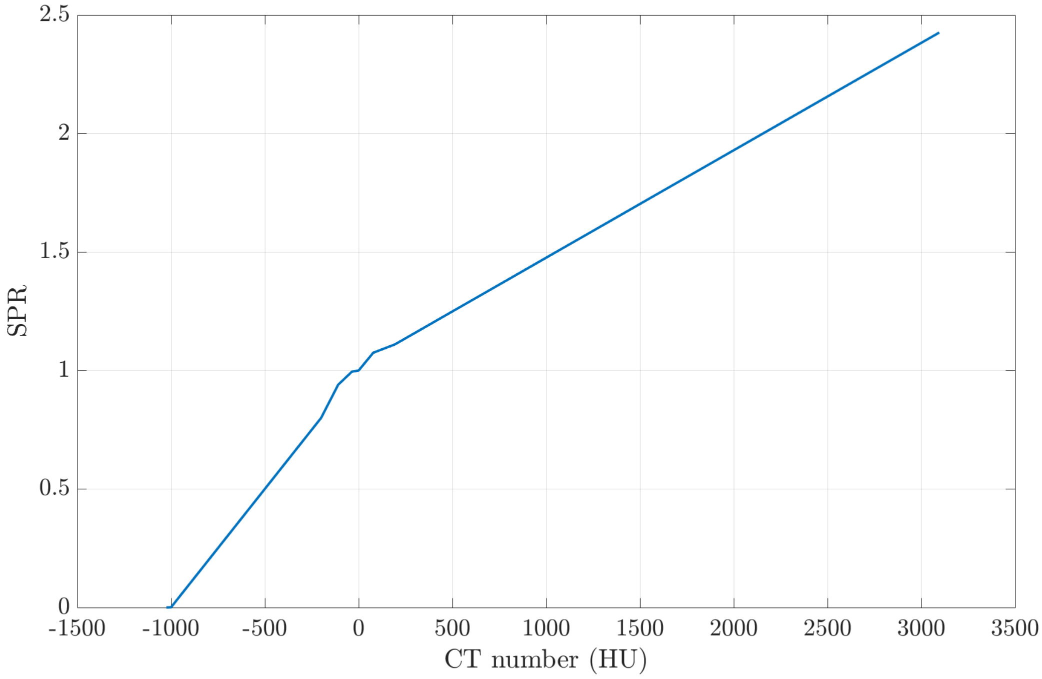

Figure 1: Single-energy CT (SECT)-based CT-number-to-stopping-power-ratio (SPR) calibration curve, or Hounsfield look-up table (HLUT), used in this study.

S2 Stopping power ratio predictions in homogeneous tissue regions

Figure 2 depicts representative regions-of-interest (ROIs) for stopping power ratio relative to water (SPR) predictions in relatively homogeneous tissue regions.

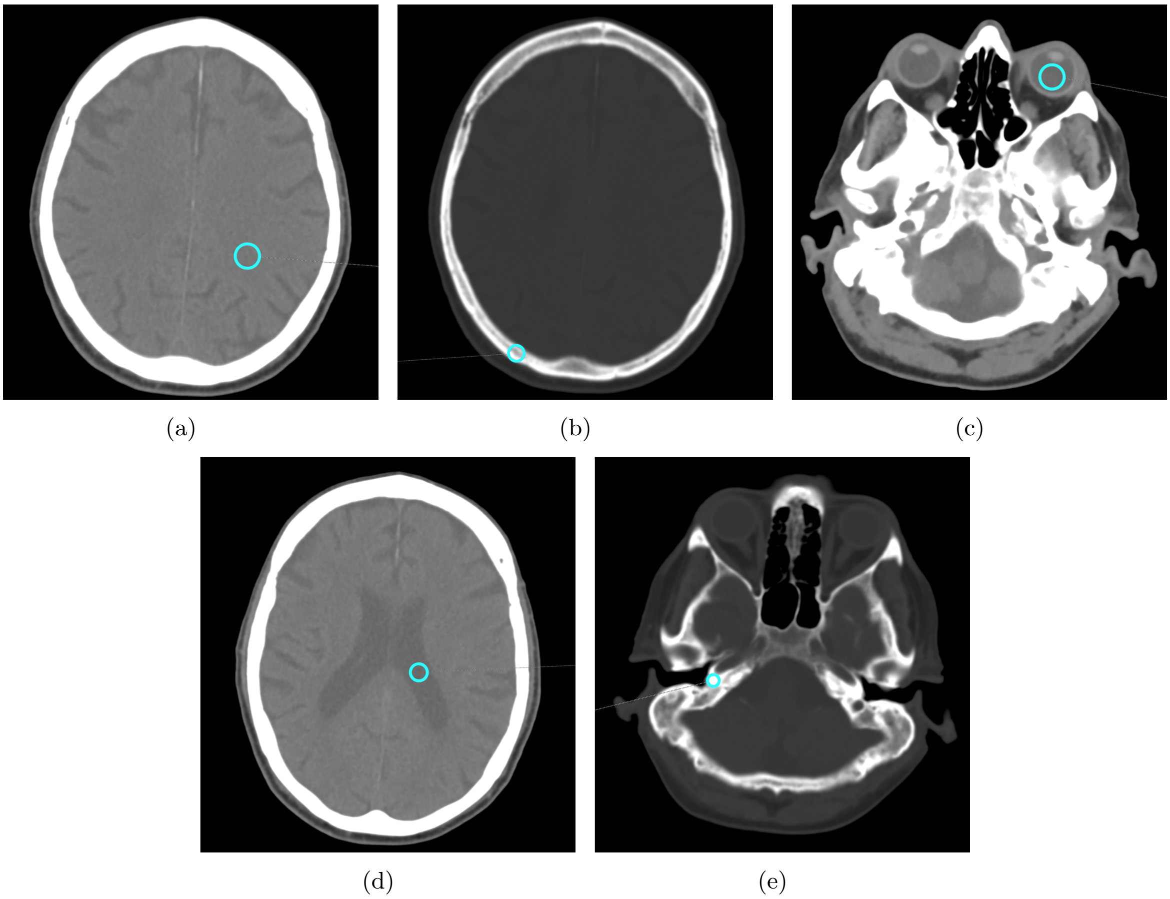

Figure 2: Representative regions-of-interest (ROIs) for a study patient (patient #1) showing brain (a), cranial bone (b), eyes (c), lateral ventricles (d), and skull base bone (e). Brain, eyes, and lateral ventricles are shown in a soft tissue window (window width: 400 HU, window center: 40 HU), whereas cranial bone and skull base bone are displayed in a bone window (window width: 1500 HU, window center: 450 HU).

S3 Analysis of dual-layer spectral CT data-based stopping power ratio prediction in head patients

SPR difference maps were generated by subtracting the SECT- from the dual-layer spectral CT (DLCT)-based SPR map and taken relative to the SPR in the DLCT-based SPR maps (DLCT was used as reference analogous to previous studies). Statistical analysis of SPR comparison between DLCT- and SECT-based methods was conducted on a per-patient basis. The absolute SPR deviation over each ROI ($\mathrm{SPR_{DLCT}} - \mathrm{SPR_{SECT}}$) was taken relative to the SPR value in the DLCT-based SPR map, respectively, similarly to Taasti et al. [1]:

$$\delta_{\mathrm{SPR}} = \frac{\Delta\mathrm{SPR}}{\mathrm{SPR_{DLCT}}} \cdot 100\ \% = \frac{\mathrm{SPR_{DLCT}} - \mathrm{SPR_{SECT}}}{\mathrm{SPR_{DLCT}}} \cdot 100\ \% \tag{1}$$

The standard deviation over n investigated slices was calculated, respectively:

$$s(\delta_{\mathrm{SPR}}) = \sqrt{\frac{\sum_{i=1}^{n}(\delta_{\mathrm{SPR},i} - \bar{\delta}_{\mathrm{SPR}})^2}{n-1}} \tag{2}$$

Here, $\overline{\delta}_{\mathrm{SPR}}$ is the arithmetic mean of the n values $\delta_{\mathrm{SPR},1}, ..., \delta_{\mathrm{SPR},n}$. The standard error of the mean (SEM) describes the variability of mean SPR difference over all patients:

$$\mathrm{SEM}(\overline{\delta}_{\mathrm{SPR}}) = \frac{s(\delta_{\mathrm{SPR}})}{\sqrt{n}} \cdot 100\ \% \tag{3}$$

To test whether the difference of the mean SPR values for SECT and DLCT was non-zero, a t-test for two paired samples was performed. The significance level was set to 5 %. The 95 %-confidence interval gave the range of values in which the population parameter lay with a probability of 95 %.

S4 Treatment plan characteristics

Table 1 summarizes the treatment plan characteristics for the four plans assessed in this study.

Table 1: Treatment plan characteristics. The table summarizes basic details for the planning target volume (PTV), prescription in relative biological effectiveness (RBE)-weighted dose, and setup for each plan. The chosen ion type is protons, helium or carbon ions with a horizontal beam angle for each plan. The couch angle is given for each beam. The number of treatment fields (i.e. couch angles) varies depending on tumor position.

	Basic details		Prescription		Setup
Plan	Tumor type	Volume of PTV (cm^3)	Dose (Gy(RBE))	Description	Couch angles (°)
A	Astrocytoma	170	50.4	28 × 1.8 Gy(RBE)	220 | 310 | 350
B	Meningioma	31	52.2	29 × 1.8 Gy(RBE)	190 | 350
C	Oligodendroglioma	182	54.0	30 × 1.8 Gy(RBE)	175 | 235
D	Pineal region tumor	73	54.0	30 × 1.8 Gy(RBE)	185 | 355

S5 Discussion of uncertainties within the study

Preceding works suggest that a systematic mean SPR bias in the irradiated volume translates directly into a mean range bias of roughly the same magnitude [2]. Similar to prior works, our study scrutinized relative ion range shifts, as opposed to absolute range predictions, and subsequent differences in dose distributions. Several uncertainty factors in SPR prediction can be classified in three different categories [3]. The first category includes imaging uncertainties (beam hardening effects [3], scanner calibration and homogeneity [4], noise [5], presence of sharp radiodensity gradients [6]). The second category involves modelling uncertainties (relative electron density (ED) and effective atomic number (EAN) accuracy, mean excitation energy (I-value) of the medium determination [7]). Uncertainties in performing DLCT data-based SPR prediction would hamper the predictions; however, ED and EAN accuracy and I-value of the medium determination were already studied and validated in previous work [8, 9, 10, 11]. Besides, several other SPR prediction methods from spectral data available in literature that were not applied in this study might further improve the predictions. The third category comprises other uncertainties (neglection of SPR energy dependence, I-value of water uncertainty). Prior studies researched into the SPR energy dependence [12] and I-value of water uncertainty [7, 13]; the optimal results found in these works were employed in this study (cf. section 2.4). The influence of the three different uncertainty categories on SPR prediction, and hence the estimated dose, depends also on the body region.

References

[1] V. T. Taasti, L. P. Muren, K. Jensen, J. B. B. Petersen, J. Thygesen, A. Tietze, C. Grau, and D. C. Hansen, "Comparison of single and dual energy CT for stopping power determination in proton therapy of head and neck cancer," *Phys. Imag. Radiat. Oncol.*, vol. 6, pp. 14–19, 2018.

[2] P. Wohlfahrt, C. Möhler, K. Stützer, S. Greilich, and C. Richter, "Dual-energy CT based proton range prediction in head and pelvic tumor patients," *Radiother. Oncol.*, vol. 125, no. 3, pp. 526–533, 2017.

[3] B. Li, H. C. Lee, X. Duan, C. Shen, L. Zhou, X. Jia, and M. Yang, "Comprehensive analysis of proton range uncertainties related to stopping-power-ratio estimation using dual-energy CT imaging," *Phys. Med. Biol.*, vol. 62, no. 17, pp. 7056–7074, 2017.

[4] H. Paganetti, "Range uncertainties in proton therapy and the role of Monte Carlo simulations," *Phys. Med. Biol.*, vol. 57, no. 11, pp. R99–R117, 2012.

[5] H. H. C. Lee, B. Li, X. Duan, L. Zhou, X. Jia, and M. Yang, "Systematic analysis of the impact of imaging noise on dual-energy CT-based proton stopping power ratio estimation," *Med. Phys.*, vol. 46, no. 5, pp. 2251–2263, 2019.

[6] T. Kairn, S. B. Crowe, P. Fogg, and J. V. Trapp, "The appearance and effects of metallic implants in CT images," *Australas. Phys. Eng. Sci. Med.*, vol. 36, no. 2, pp. 209–217, 2013.

[7] V. De Smet, R. Labarbe, F. Vander Stappen, B. Macq, and E. Sterpin, "Reassessment of stopping power ratio uncertainties caused by mean excitation energies using a water-based formalism," *Med. Phys.*, vol. 45, no. 7, pp. 3361–3370, 2018.

[8] F. K. Faller, S. Mein, B. Ackermann, J. Debus, W. Stiller, and A. Mairani, "Pre-clinical evaluation of dual-layer spectral computed tomography-based stopping power prediction for particle therapy planning at the Heidelberg Ion Beam Therapy Center," *Phys. Med. Biol.*, vol. 65, no. 9, p. 095007, 2020.

[9] G. Landry, F. Dörringer, S. Si-Mohamed, P. Douek, J. F. P. J. Abascal, F. Peyrin, I. P. Almeida, F. Verhaegen, I. Rinaldi, K. Parodi, and S. Rit, "Technical Note: Relative proton stopping power estimation from virtual monoenergetic images reconstructed from dual-layer computed tomography," *Med. Phys.*, vol. 46, no. 4, pp. 1821–1828, 2019.

[10] K. Mei, S. Ehn, M. Oechsner, F. K. Kopp, D. Pfeiffer, A. A. Fingerle, F. Pfeiffer, S. E. Combs, J. J. Wilkens, E. J. Rummeny, and P. B. Noël, "Dual-layer spectral computed tomography: measuring relative electron density," *Eur. Radiol. Exp.*, vol. 2, no. 1, pp. 1–9, 2018.

[11] S. Ohira, H. Washio, M. Yagi, T. Karino, K. Nakamura, Y. Ueda, M. Miyazaki, M. Koizumi, and T. Teshima, "Estimation of electron density, effective atomic number and stopping power ratio using dual-layer computed tomography for radiotherapy treatment planning," *Physica Medica*, vol. 56, pp. 34–40, 2018.

[12] T. Inaniwa and N. Kanematsu, "Effective particle energies for stopping power calculation in radiotherapy treatment planning with protons and helium, carbon, and oxygen ions," *Phys. Med. Biol.*, vol. 61, no. 20, pp. N542–N550, 2016.

[13] E. Bär, P. Andreo, A. Lalonde, G. Royle, and H. Bouchard, "Optimized I-values for use with the Bragg additivity rule and their impact on proton stopping power and range uncertainty," *Phys. Med. Biol.*, vol. 63, no. 16, p. 165007, 2018.
